# Candida Colonization Index in Patients Admitted to an ICU

**DOI:** 10.3390/ijms12107038

**Published:** 2011-10-20

**Authors:** Giuseppina Caggiano, Filomena Puntillo, Caterina Coretti, Mariateresa Giglio, Ilaria Alicino, Fabio Manca, Francesco Bruno, Maria Teresa Montagna

**Affiliations:** 1 Department of Biomedical Science and Human Oncology–Hygiene Section, University of Bari “Aldo Moro”, Piazza G. Cesare no. 11, 70124 Bari, Italy; E-Mails: catiacoretti@yahoo.it (C.C.); montagna@igiene.uniba.it (M.T.M.); 2 Department of Emergency and Organ Transplantation-Anaesthesia and Intensive Care Unit, University of Bari “Aldo Moro”, Piazza G. Cesare no. 11, 70124 Bari, Italy; E-Mails: fpuntillo@rianima.uniba.it (F.P.); mariateresagiglio@libero.it (M.G.); ilaria.alicino@libero.it (I.A.); bruno@rianima.uniba.it (F.B.); 3 Department of Historical and Geographic Science–Statistic-University of Bari “Aldo Moro”, Piazza Umberto I no. 1, 70124 Bari, Italy; E-Mail: fmanca@dipstogeo.uniba.it

**Keywords:** *Candida* colonization, Colonization index, *Candida* mannan, *Candida* anti-mannan antibodies

## Abstract

Multiple-site colonization with *Candida* spp. is commonly recognized as a risk factor for invasive fungal infection in critically ill patients. We carried out a study to determine the relationship between *Candida* colonization and invasive infection in neurological patients admitted to an ICU. At admission (T0) and every three days for two weeks, different samples (pharynx swab, tracheal secretions, stomach contents, *etc*.) were collected for mycological surveillance. *Candida* mannan antigen and *Candida* anti-mannan antibodies were assayed. The Colonization Index (CI) and Corrected Colonization Index were calculated for each time point. Of all patients 70% was already colonized by *Candida* spp. at T0 and six of them had CI ≥ 0.5. Three patients developed candidemia; they had CI ≥ 0.5 before infection. Positive values of *Candida* mannan antigen and anti-mannan antibodies were found only in the patients with candidemia. The sensitivity and specificity of the *Candida* mannan test were 66.6% and 100%, respectively, while the sensitivity and specificity of the anti-mannan antibody test were 100%. In accordance with other authors, we find the surveillance cultures are useful to monitor the *Candida* colonization in ICU patients. In addition, the sequential observation of anti-mannan antibodies could contribute to early diagnosis of candidiasis more than *Candida* mannan antigen in immunocompetent patients.

## 1. Introduction

Invasive fungal infections are an important cause of morbidity and mortality in immunocompromised subjects such as patients with severe neutropenia or cancer and in patients admitted to ICU, intensive care of neonatology or pediatric intensive care units [1–3]. Currently, chemotherapy, intravascular catheter, prolonged stays in ICUs, immunosuppressive therapy for organ transplantation, abdominal surgery and *Candida* colonization are recognized as risk factors for invasive mycoses [4,5]. In particular, *Candida* colonization, originating from the endogenous flora that develops within the gastrointestinal tract, seems to be the first step towards severe infection [6–8]. In fact, during the 1980s, Wey *et al*. [9] identified *Candida* colonization as an independent risk factor for candidemia. Actually, multiple-site colonization with *Candida* spp. is commonly recognized as a major risk factor for invasive fungal infection in critically ill patients and the colonization density could be a predictive value for the diagnosis of systemic candidiasis [10–12], nevertheless the distinction between colonization and infection is often difficult [13]. Although multiple surveillance cultures are often performed daily for critically ill patients, the clinical importance of positive *Candida* spp. cultures is difficult to define. *Candida* colonization is present in 5–15% of patients but it can achieve peaks of 50–86% and an invasive infection can develop in 5–30% of colonized patients [14,15]. The death risk in patients with distinct colonized body sites is similar to patients with proven *Candida* invasive infection [15]. Pittet *et al*. [16] demonstrated that *Candida* colonization assessed by systematic screening helps to predict infections in critically ill patients. They defined the Colonization Index (CI) and the Corrected Colonization Index (CCI); CI value ≥ 0.5 and CCI ≥ 0.4 were considered thresholds to distinguish patients with *Candida* infection [16]. The choice of surveillance samples to evaluate the CI and CCI has not been established, but it would be appropriate to use samples in which the isolation of fungi is higher (pharynx swab, tracheal aspirate, rectal swab, *etc*.). This prospective study aimed at evaluating the relationship between *Candida* colonization and invasive infection in neurosurgical patients admitted to an ICU. Moreover, the usefulness of *Candida* mannan antigen and *Candida* anti-mannan antibodies as markers of infection was also analyzed.

## 2. Materials and Methods

This study was carried out in the Intensive Care Unit of the University Hospital of Southern Italy (UHSI) during January–December 2008. UHSI is a 1300-bed teaching hospital with about 500 admissions per year to the ICU.

The eligible patients were neurosurgical patients of age >18 years, admitted to the ICU because of traumatically or post-operative complications and without documented *Candida* infection at admission. Patients who stayed in the ICU for <7 days were excluded from the study.

A total of 51 patients met the criteria for inclusion and were prospectively observed for our study. At admission (T0) to the ICU and every three days for 2 weeks (T3, T6, T9, T12, T15) the following samples were collected for mycological surveillance: pharynx swab, tracheal secretions, stomach contents, rectal swab, armpit skin swab, urine, blood, serum for *Candida* mannan antigen and *Candida* anti-mannan antibodies determination.

At each time interval, the CI and CCI were calculated.

The study was approved by the Ethics Committee of Hospital and informed consent was obtained from the patients or their representative.

### 2.1. Microbiological Methods

Every biological specimen was cultured on Sabouraud dextrose agar with 0.05% chloramphenicol (Oxoid S.p.A.) and chromogenic medium plates (CHROMagar TM Candida Medium, Becton Dickinson, Germany) to identify polymicrobial cultures. All plates were incubated at 36 °C (±1) and the cultures were examined daily for growth for up to six days before declaring them negative. Blood cultures were performed using a lyses centrifugation system (Isolator^®^ DuPont Co. Wilmington–Delaware). The isolates were identified by analysis of biochemical patterns using the ID 32C and Vitek II systems (bioMérieux, France).

The serum was frozen at −20 °C and once a week *Candida* mannan antigen and anti-mannan antibodies were quantified.

The detection *of Candida* mannan antigen was measured using a Platelia ELISA system (Platelia^®^ Candida Ag EIA; BIO-RAD, France) according to the manufacturer’s instructions. The cut-off values recommended by the manufacturer for interpretation of results were ≥0.5 ng/mL as positive, ranging from 0.25–0.49 ng/mL as intermediate and <0.25 ng/mL as negative. *Candida* anti-mannan antibodies were measured using the Platelia^®^ Candida Ab/Ac/Ak EIA (BIO-RAD, France) according to the manufacturer’s instructions. The cut-off values for interpretation of results were >10 AU/mL as positive, 5–10 AU/mL as intermediate and <5 AU/mL as negative. Each serum was tested in duplicate.

The antifungal susceptibility of yeast strains responsible for infection was evaluated using the E-test method. E-test strips for amphotericin B (AMB), anidulafungin (AND), caspofungin (CSP), itraconazole (ITC), posaconazole (PSC), voriconazole (VRC) (concentrations ranging from 0.002 to 32 mg/L) and for fluconazole (FLC) (from 0.16 to 256 mg/L) were obtained from AB BIODISK (Solna, Sweden) The E-test assay was performed on RPMI 1640 agar plates (Biolife, Milan, Italy) following the manufacturer’s instructions. The plates were incubated at 35 °C and read after 24 h. The Minimal Inhibitory Concentration (MIC) was taken as the drug concentration at which the border of the elliptical inhibition zone intersected the scale on the antifungal test strip. The growth of microcolonies within this inhibition zone was disregarded.

Interpretative criteria were those suggested by the Clinical and Laboratory Standards Institute (CLSI): AND and CSP, Susceptible ≤ 2 mg/L, Non-Susceptible > 2 mg/L; FLC, S < 8 mg/L, Susceptible Dose-Dependent 16–32 mg/L, R > 64 mg/L; ITC, Susceptible ≤ 0.125 mg/L, Susceptible Dose-Dependent 0.25–0.5 mg/L, Resistant ≥ 1 mg/L; VRC, Susceptible ≤ 1 mg/L, Susceptible Dose-Dependent 2 mg/L, Resistant ≥ 4 mg/L [17]. Interpretive criteria for AMB and PSC have not been established; so in accordance with literature data we selected a breakpoint of >1.0 mg/L to define an isolate as AMB Resistant and VRC breakpoints were applied to the PSC MIC value [18,19].

### 2.2. Definitions

*Candida* colonization was defined as repeated growth of yeasts from at least two different sites. According to Pittet’s definitions, the CI was defined as the ratio of the number of distinct non-blood body sites colonized by *Candida* to the total number of body sites cultured. The CCI was defined as the product of the CI multiplied by the ratio of the number of distinct body sites showing heavy growth to the total of distinct body sites growing *Candida* [17]. Patients with CI ≥ 0.5 and CCI ≥ 0.4 were considered heavily colonized. Patients were considered infected if a positive *Candida* spp. culture from blood or other sites normally sterile was documented.

### 2.3. Statistical Investigation

The data analysis was executed with the statistical software SPSS (Statistical Package for Social Sciences). The statistical model used to arrive at the significance of the averages during the study period and among the different specimens was the ANOVA model (variance analysis), it allowed to calculate the F test and the *p* value < 0.05. The technique of multiple comparison with the Tukey HSD model to analyze the significant differences among groups was used. The tests gave information on which pairs of averages were significantly different.

## 3. Results

A total of 51 patients were enrolled, 30 male and 21 female, of median age 58 ± 18 years (range 18–83 years). They were monitored for 15 days. The more frequent predisposing factors for *Candida* infection were the presence of central venous catheter (100% of patients), antibiotic therapy (80.3%), diabetes (21.5%) and corticosteroid therapy (13.7%). None of the patients received antifungal prophylaxis or antifungal treatment previous to ICU admission. The SOFA score at admission was on average 6.98 ± 2.64.

### 3.1. Colonization Index

**Figure 1:**
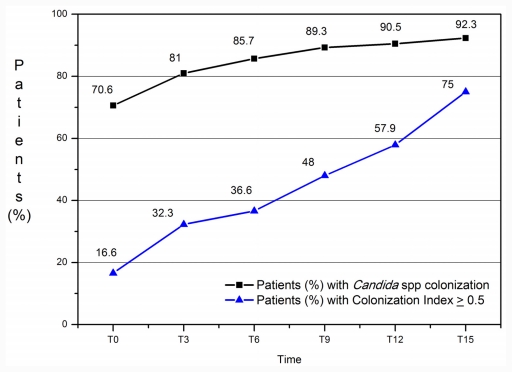
Percentage of patients with *Candida* spp. colonization and Colonization Index ≥ 0.5 during the study period.

Figure 1 shows the percentage of patients with *Candida* spp. colonization and CI ≥ 0.5 during residence in the ICU. Overall, at admission, 70.6% of patients were already colonized and the percentage increased progressively to 92% at T15. Twenty-nine patients were colonized by one species, 12 by two species; three different *Candida* species were isolated in one case. Considering the species: *C. albicans* was isolated in 76.2% of samples collected for mycological surveillance, *C. glabrata* in 23.8%, *C. krusei* in 19%, *C. parapsilosis* in 9.5%, *C. tropicalis* and *C. kefyr* in 4.7%, and *C. inconspicua* in 2.4%. At T0, 16.6% of colonized patients had CI ≥ 0.5 and this percentage increased during the observation period up to 75% at T15.

The colonization trend in each examined body site demonstrates a major colonization in the stomach, trachea and pharynx *versus* skin and urine on each day of observation (Anova analysis, *F* = 7.212; *p* < 0.003). The CCI mean value was ≥ 0.4 in all observations with the exception of T0.

**Figure 2:**
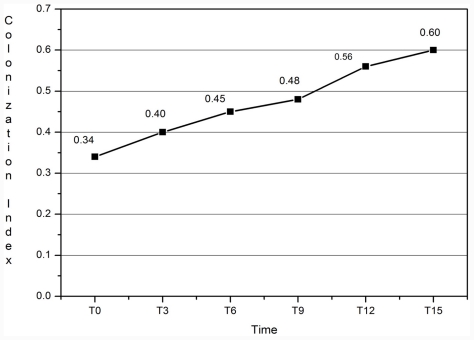
Colonization Index media observed during the study period.

The CI media increased progressively during the observation period (Figure 2) (*p* = 0.003, 95% Conf Interval D: 25.5–47.1).

During our study, three patients developed invasive infection by *C. albicans* on the 12th, 13th and 15th day, respectively after admission to the ICU; all three patients had CI ≥ 0.5 before candidemia. The features of the three patients with candidal bloodstream infection are documented in the Table 1. The three *C. albicans* strains resulted susceptible to tested antifungal drugs: AND range 0.016–0.03 mg/L; CSP 0.25–1 mg/L; PSC 0.03–0.125 mg/L; VRC 0.008–0.016 mg/L; ITC 0.06–0.125 mg/L; FLC 0.125–0.5 mg/L; AMB 0.25–0.5 mg/L.

### 3.2. Candida Mannan Antigen and Anti-Mannan Antibodies

The circulating *Candida* mannan and anti-mannan antibodies tests resulted negative in all patients without invasive infection. In the three infected patients, the anti-*mannan* antibodies resulted positive 4, 5 and 7 days earlier than isolation of fungi in blood cultures, respectively. The *Candida* mannan antigen failed as an early marker of infection, becoming positive in two cases 1 and 4 days later than blood cultures resulted positive and in one case, it was always negative despite the infection (Table 1).

In patients with candidemia the sensitivity and specificity of the *Candida* mannan test were 66.6% and 100% respectively, while the sensitivity and specificity of the anti-mannan antibodies tests were 100%.

## 4. Discussion

It is well known that ICU patients are at high risk of fungal infection. Among the different risk factors *Candida* colonization is an important one. In our study we observed only immunocompetent patients with neurosurgical complications and many of them were already colonized by *Candida* spp. on admission to the ICU, but only 16% had CI ≥ 0.5. The percentage of colonized patients increased significantly on the 6th day of their stay in the ICU. The percentage of patients with CI ≥ 0.5 increased significantly during the period T0–T15. In contrast, 90% of patients not colonized at T0 remained not colonized throughout their stay in the ICU. In this study invasive methods to assess for colonization were used such as aspiration of tracheal secretions and stomach contents; stomach and pharynx remained the most colonized body sites in the period from T0 to T15. During the observation period three patients developed *Candida* bloodstream infection, they presented CI ≥ 0.5. These results are in accordance to data of other researchers that show *Candida* colonization often precedes an invasive fungal infection and that the risk is correlated to CI [6,20]. Dubau *et al*. [21] demonstrated that antifungal prophylaxis in 35 heavily colonized patients (CI ≥ 0.5) could reduce the density of colonization, prevent infection and improve prognosis.

Despite continuing progress, the diagnosis of *Candida* invasive infection still presents problems because the conventional diagnostic is often insensitive and time-consuming. Some procedures (e.g., biopsies) require aggressive approaches that are not compatible with the critical conditions of ICU patients. Several assays have been developed to help the diagnosis of deep mycoses, among them tests for the detection of *Candida* mannan antigen and anti-mannan antibodies [22–25]. Studies carried out on mannan have demonstrated its role as an important modulator of innate and adaptive immunity; mannan induces a strong antibody response. The detection of mannan is quite specific for the diagnosis of invasive candidiasis, although this technique requires frequent sampling due to the rapid clearance of these antigens from the blood. Recent studies have demonstrated good diagnostic efficacy associating this assay with the search for anti-mannan antibodies in critical, but not in immunocompromised patients [26]. Retrospective studies on serum samples from 130 patients with candidemia and 150 serum samples from patients without fungemia showed that the combined detection of mannan and anti-mannan antibodies can increase specificity and sensitivity in the diagnosis of *Candida* spp. deep infection [27,28].

In our study all sera of patients without deep *Candida* infection resulted negative to *Candida* mannan and anti-mannan antibodies tests, while they resulted positive in patients with candidemia. Besides, as also noted by other authors [26] we observed an inverse relationship between antigen and antibodies levels; in fact anti-mannan antibodies levels increased before notification of yeasts in blood culture, and mannan antigen resulted positive.

## 5. Conclusions

Some important information can be derived from this study. The use of more invasive methods to assess for colonization including gastric and tracheal samples could be very useful to increase the probability to meet colonized patients. It is important to underline that, in immunocompetent patients, the anti-mannan antibodies levels may increase precociously and mask the circulating *Candida* mannan. The sensitivity of the tests is enhanced by regular serological sampling, thus, detection of anti-*Candida* antibodies could be a useful contribution to early diagnosis of invasive infection, but further studies should be undertaken in order to better confirm these data. Further, it is of particular interest that CI increases significantly after the six days of ICU stay and consequently *Candida* invasive infection is more likely to occur. At T15, among patients with CI ≥ 0.5 the percentage of subjects that developed candidemia exceeded 30%. Thus, our experience suggests that monitoring CI could be helpful in identifying patients at risk of invasive fungal infection. In addition, complementing this with anti *Candida* antibodies detection in immunocompetent patients with CI ≥ 0.5 increases the positive predictivity for infection allowing an early diagnosis of candidemia. Unfortunately, to date prospective surveillance studies of fungal colonization in the ICU are still limited and they use different samples for the mycological follow-up of patients.

Although further studies are needed to compare, standardize and eventually confirm these data it is possible to support that monitoring for colonization with *Candida* species in patients recovered to ICU for >7 days may offer opportunity for early intervention for prevention of candidemia.

## Figures and Tables

**Table 1:** Features of the three patients with candidal bloodstream infection.

	Pt 1	Pt 2	Pt 3
Sex	M	F	F
Age	26	79	52
Sofa	8	8	8
WBC	20 × 10^3^/mm^3^	7.5 × 10^3^/mm^3^	15 × 10^3^/mm^3^
MV (days)	27	50	15
Admission days in ICU (no.)	36	57	15
CI (the day of candidemia)	0.5	0.7	0.5
Mannan Ag	+	///	+
Ag Circulation	4 d P	///	1 d P
Ab-anti *Candida*	14.5 UA/mL	12.8 UA/mL	11.20 UA/mL
Ab Circulation	7 d B	4 d B	5 d B
Outcome	living	died	living

M = Male; F = Female; MV = Mechanical Ventilation; + = Circulation of mannan Ag; /// = lack of mannan Ag; d P = day post candidemia; d B = days before candidemia; CI = Colonization Index at the moment of candidaemia.

## References

[1] Caggiano G, Iatta R, Laneve A, Manca F, Montagna MT (2008). Observational study on candidaemia at a university hospital in southern Italy from 1998 to 2004. Mycoses.

[2] Zaoutis TE, Prasad PA, Localio AR, Coffin SE, Bell LM, Walsh TJ, Gross R (2010). Risk factors and predictors for candidemia in pediatric intensive care unit patients: implications for prevention. Clin. Infect. Dis.

[3] Pfaller MA, Diekema DJ (2007). Epidemiology of invasive candidiasis: A persistent public health problem. Clin. Microbiol. Rev.

[4] Bouza E, Munoz P (2008). Epidemiology of candidemia in intensive care units. Int J Antimicrob Agents.

[5] Eggimann P, Garbino J, Pittet D (2003). Epidemiology of *Candida* species infections in critically ill non immunosuppressed patients. Lancet Infect. Dis.

[6] Klempp-Selb B, Rimek D, Kappe R (2000). Karyotiping of *Candida albicans* and *Candida glabrata* from patients with *Candida* sepsis. Mycoses.

[7] Singhi S, Rao DS, Chakrabarti A (2008). *Candida* colonization and candidemia in a pediatric intensive care unit. Pediatr. Crit. Care Med.

[8] Ruping MJ, Vehreschild JJ, Cornely OA (2008). Patients at high risk of invasive fungal infection: when and how to treat. Drugs.

[9] Wey SB, Mori M, Pfaller MA, Woolson RF, Wenzel RP (1989). Risk factors for hospital-acquired candidemia. A matched case-control study. Arch. Int. Med.

[10] Agvald-Ohman C, Klingspor L, Hjelmqvist H, Edlund C (2008). Invasive candidiasis in long-term patients at a multidisciplinary intensive care unit: Candida colonization index, risk factors, treatment and outcome. Scand. J. Infect. Dis.

[11] Almirante B, Rodríguez D, Park BJ, Cuenca-Estrella M, Planes AM, Almela M, Mensa J, Sanchez F, Ayats J, Gimenez M (2005). Epidemiology and predictors of mortality in cases of Candida bloodstream infection: Results from population-based surveillance, Barcelona, Spain, from 2002 to 2003. J. Clin. Microbial.

[12] Charles PE, Dalle F, Aube H, Doise JM, Quenot JP, Aho LS, Chavanet P, Blettery B (2005). *Candida* spp. colonization significance in critically ill medical patients: A prospective study. Intensive Care Med.

[13] Paphitou NI, Ostrosky-Zeichner L, Rex JH (2005). Rules for identifying patients at increased risk for candidal infections in the surgical intensive care unit: Approach to developing practical criteria for systematic use in antifungal prophylaxis trials. Med. Mycol.

[14] Petri MG, Konig J, Moecke HP (1997). Epidemiology of invasive mycosis in ICU patients: A prospective multicenter study in 435 non-neutropenic patients. Intensive Care Med.

[15] Slotman G, Shapiro E, Moffa S (1994). Fungal sepsis: multisite colonization *versus* fungemia. Am. Surg.

[16] Pittet D, Monod M, Suter PM, Frenk E, Auckenthaler R (1994). *Candida* colonization and subsequent infections in critically ill surgical patients. Ann. Surg.

[17] Clinical and Laboratory Standard Institute (2008). Reference Method for Broth Dilution Antifungal Susceptibility Testing of Filamentous Fungi.

[18] Pfaller MA, Diekema DJ, Jones RN, Sader HS, Fluit AC, Hollis RJ, Messer SA, SENTRY Participant Group (2001). International surveillance of bloodstream infections due to *Candida* species: frequency of occurrence and *in vitro* susceptibilities to fluconazole, ravuconazole, voriconazole of isolates collected from 1997 through 1999 in the SENTRY antimicrobial surveillance program. J. Clin. Microbiol.

[19] Diekema DJ, Messer SA, Boyken LB, Hollis RJ, Kroeger J, Tendolkar S, Pfaller MA (2009). In vitro activity of seven systemically active antifungal agents against a large global rare Candida species as determined by CLSI broth microdilution methods. J. Clin. Microbiol.

[20] Garbino J, Lew DP, Romand JA, Hugonnet S, Auckenthaler R, Pittet D (2002). Prevention of severe *Candida* infections in non-neutropenic, high-risk, critically ill patients. A randomized, double-blind, placebo-controlled trial in SDD-treated patients. Intensive Care Med.

[21] Dubau B, Triboulet C, Winnock S (2001). Utilisation pratique de l’index de colonisation. Ann. Fr. Anesth. Reanim.

[22] Ellis M, Al-Rmandi B, Bernsen R, Kristensen J, Alizadeh H, Hedstrom U (2009). Prospective evaluation of mannan and anti-manna antibodies for diagnosis of invasive Candida infections in patients with neutropenic fever. J. Med. Microbiol.

[23] Laín A, Elguezabal N, Moragues MD, García-Ruiz JC, Del Palacio A, Pontón J (2008). Contribution of serum biomarkers to the diagnosis of invasive candidiasis. Expert Rev. Mol. Diagn.

[24] Pontón J (2009). Usefulness of biological markers in the diagnosis of invasive candidiasis. Rev. Iberoam. Micol.

[25] Prella M, Bille J, Pugnale M, Duvoisin B, Cavassini M, Calandra T, Marchetti O (2005). Early diagnosis of invasive candidiasis with mannan antigenemia and antimannan antibodies. Diagn. Microbiol. Infect. Dis.

[26] Shea YR, Murray P, Baron EJ, Jorgensen J, Landry ML, Pfaller M (2007). Algorithms for Detection and Identification of Fungi. Manual of Clinical Microbiology.

[27] Sendid B, Poirot JL, Tabouret M, Bonnin A, Caillot D, Camus D, Poulain D (2002). Combined detection of mannanaemia and antimannan antibodies as a strategy for the diagnosis of systemic infection caused by pathogenic Candida species. J. Med. Microbiol.

[28] Sendid B, Caillot DE, Baccouch-Humbert B, Klingspor L, Grandjean M, Bonnin A, Poulain D (2003). Contribution of the Platelia *Candida* Specific Antibody and Antigen tests to early diagnosis of systemic *Candida tropicalis* infection in neutropenic adults. J. Clin. Microbiol.

